# The Association of Symptom Burden With the Risk for Falls in Community-Dwelling Older Adults.

**DOI:** 10.1093/geroni/igaf122.2153

**Published:** 2025-12-31

**Authors:** Michelle McKay, Paul Bernhardt, Melissa O’Connor, Jessica Yarnall, Suzanne Leveille

**Affiliations:** Villanova University, Villanova, Pennsylvania, United States; Villanova University, Villanova, Pennsylvania, United States; Hunter College, New York, New York, United States; Villanova University, Villanova, Pennsylvania, United States; University of Massachusetts Boston, Boston, Massachusetts, United States

## Abstract

One in four older adults (≥65 years) suffer a fall each year. However, falls are not a part of normal aging and are often preventable. An underexplored area of research is the role of symptom burden as a risk for falls in older adults. The purpose of this study was to determine whether symptom burden, measured as symptom severity classes based on subjective severity ratings of pain, balance, endurance, weakness, sleep, hearing, anxiety, and vision, was associated with time to first fall and experiencing a fall within one year. A secondary analysis of the MOBILIZE Boston dataset, which examined novel risk factors for falls in a population-based cohort of older adults (n = 765), was conducted. Initially, latent class analysis identified 4 symptom severity classes across all symptoms (mild, moderate, moderate-severe, severe). Descriptive statistics, cox proportional hazards modeling (hazard ratios, HR), and logistic regression (odds ratios, OR) were used for further analysis of symptom severity classes and falls. In the unadjusted Cox PH models for time to first fall, the hazard of falling was found to increase with each symptom severity level [HR = 1.44 (1.30, 1.61)]. Similar results were noted with logistic regression modeling; each level increase in symptom severity class was associated with an increased risk for falls within the first year [OR = 1.91 (1.55, 2.38)]. Symptom burden, as a subjective measure, may enable identification of older adults at risk for falls. Using a symptom-based treatment approach may lead to fewer falls and the associated negative consequences.

